# Touched by loneliness—how loneliness impacts the response to observed human touch: a tDCS study

**DOI:** 10.1093/scan/nsab122

**Published:** 2021-12-31

**Authors:** Nira Saporta, Leehe Peled-Avron, Dirk Scheele, Jana Lieberz, René Hurlemann, Simone G Shamay-Tsoory

**Affiliations:** School of Psychological Sciences, University of Haifa, Haifa 3498838, Israel; School of Psychological Sciences, University of Haifa, Haifa 3498838, Israel; Division of Medical Psychology, Department of Psychiatry and Psychotherapy, University Hospital Bonn, Bonn 53105, Germany; Department of Psychiatry, School of Medicine & Health Sciences, University of Oldenburg, Oldenburg 26129, Germany; Division of Medical Psychology, Department of Psychiatry and Psychotherapy, University Hospital Bonn, Bonn 53105, Germany; Department of Psychiatry, School of Medicine & Health Sciences, University of Oldenburg, Oldenburg 26129, Germany; Research Center Neurosensory Science, University of Oldenburg, Oldenburg 26129, Germany; School of Psychological Sciences, University of Haifa, Haifa 3498838, Israel

**Keywords:** loneliness, affective touch, tDCS, inferior frontal gyrus

## Abstract

Lonely people often crave connectedness. However, they may also experience their environment as threatening, entering a self-preserving state that perpetuates loneliness. Research shows conflicting evidence about their response to positive social cues, and little is known about their experience of observed human touch. The right inferior frontal gyrus (rIFG) is part of an observation–execution network implicated in observed touch perception. Correlative studies also point to rIFG’s involvement in loneliness. We examined the causal effect of rIFG anodal transcranial direct current stimulation on high- and low-loneliness individuals observing human touch. In a cross-over design study, 40 participants watched pictures of humans or objects touching or not touching during anodal and sham stimulations. Participants indicated whether pictures contained humans or objects, and their reaction time was measured. Results show that the reaction time of low-loneliness individuals to observed human touch was significantly slower during anodal stimulation compared to high-loneliness individuals, possibly due to them being more emotionally distracted by it. Lonely individuals also reported less liking of touch. Our findings support the notion that lonely individuals are not drawn to positive social cues. This may help explain the perpetuation of loneliness, despite social opportunities that could be available to lonely people.

## Introduction

Human beings are social by nature. As humans, we have a fundamental ‘need to belong’ (Baumeister and Leary, 1995). When this need is not fulfilled, people experience loneliness, a subjective distressing experience described as a chronic feeling of perceived social isolation (Weiss, 1973) or an unpleasant experience that occurs when a person’s network of social relations is deficient (Perlman and Peplau, 1981).

From an evolutionary standpoint, loneliness was postulated to be adaptive since it serves as an aversive signal, which motivates people to reconnect, much like hunger motivates people to seek food (Cacioppo *et al.*, 2014a; Tomova *et al.*, 2020). However, loneliness can also become chronic, and it is receiving increasing academic and public attention since it was shown to have harmful effects on physical and mental health (Holt-Lunstad *et al.*, 2015; Lim *et al.*, 2020).

If loneliness is an aversive signal that is supposed to motivate toward reconnection, how come certain people experience it in a perpetuating manner? Evolutionary theory suggests that chronic loneliness will induce biases such that lonely people believe they cannot rely on their environment for protection and help. Loneliness will therefore activate neural, neuroendocrine and behavioral responses aiming to protect the individual, inducing a self-preserving state. These include an activation of the hypothalamic–pituitary–adrenocortical axis, altered vascular activity and increased anxiety, vigilance and withdrawal (Cacioppo and Hawkley, 2009; Cacioppo *et al.*, 2014b, 2015a; Cacioppo and Cacioppo, 2018). In congruence with this perspective, lonely people were shown to have a bias toward attending to social threats such as negative facial expressions, negative social situations and negative vocal tone (Cacioppo and Hawkley, 2009; Cacioppo *et al.*, 2016; Spithoven *et al.*, 2017; Shin and Kim, 2019). Thus, chronic loneliness may drive people away from regaining social connectedness.

How would such a self-preserving state impact lonely people’s response to positive social cues? The literature shows conflicting evidence. On the one hand, lonely people displayed greater incidental memory of both positive and negative social information, heightened attention to both positive and negative vocal tones and heightened accuracy in identifying facial expressions (Gardner *et al.*, 2005), increased attention to smiling faces (DeWall *et al.*, 2009), greater ability to detect real and fake smiles (Bernstein *et al*., 2008) and increased attention toward warm faces (Saito *et al.*, 2020). In contrast, other studies show reduced responses of lonely individuals to positive social stimuli (Lieberz *et al.*, 2021; Saporta *et al.*, 2021b). Lonely people exhibited reduced neural activity in areas related to reward (ventral striatum) while viewing pleasant interactions, compared to low-loneliness individuals (Cacioppo *et al.*, 2009). This was interpreted to signify that lonely people experience less enjoyment from viewing positive interactions. Moreover, participants with higher levels of chronic loneliness reported less craving for social contact in response to positive social cues and showed a muted response in the substantia nigra and the ventral tegmental area to social cues after acute isolation (Tomova *et al.*, 2020).

One of the most powerful positive social cues that exist is social touch. Touch encompasses a large variety of behaviors that involve physical contact. In recent years it has regained scientific interest as a subject of investigation on the neural, physiological and cognitive levels (Gliga *et al.*, 2019). Touch is the earliest sense to develop, providing us with means of contact with the external world (Gottlieb, 1971; Barnett, 1972). Acts of touch are essential to social interactions and convey feelings and thoughts in interpersonal communication (Hertenstein *et al.*, 2009). Affectionate touch promotes relational, psychological and physical well-being (Gallace and Spence, 2016; Jakubiak and Feeney, 2017; Cascio *et al.*, 2019; Field, 2019).

Interestingly, despite the critical importance of human touch to social connectedness, very little is known about the way lonely people experience and perceive human social touch. It was found that holding a warm thermos reduces loneliness (Murphy and Standing, 2014) and that people reported feeling less neglected after being briefly touched (Heatley Tejada *et al.*, 2020). However, no study to date specifically examined lonely people’s reaction to observing stimuli depicting positive human social touch.

Notably, studies show that the right inferior frontal gyrus (rIFG) is involved in the neural processing of touch. It has been shown to be involved in tactile object recognition and localization (Reed *et al.*, 2005), as well as the neural processing of touch stimulation (May *et al.*, 2014). More broadly, the IFG is central to the inferior frontoparietal observation–execution (OE) network, which is critical to forming social connections (Cacioppo and Cacioppo, 2012; Peled-Avron *et al.*, 2019). We have previously reported that anodal transcranial direct current stimulation (tDCS) of the rIFG was associated with emotional responses to observed social touch, which supports the notion that the rIFG plays a key role in the perception of observed tactile stimuli (Peled-Avron *et al.*, 2019).

Intriguingly, the IFG, and in particular the rIFG, seems to also be involved in loneliness. The particulars of its involvement are yet unclear. In a structural study it was demonstrated that increased loneliness was correlated with a decrease in fractional anisotropy of white matter tracts that are linked to IFG, the anterior insula (AI) and the temporoparietal junction (TPJ), suggesting an impaired connectivity of these areas in the ventral attention network (Tian *et al.*, 2014). Lesion to the right PFC, including superior frontal gyrus, right middle and inferior frontal gyrus, and right insula were significantly associated with decreased loneliness scores suggesting that in intact brains the activity of these areas is related to increased loneliness (Cristofori *et al.*, 2019). In line with the structural studies, functional neuroimaging studies have shown that lonely people have different neural response patterns to social stimuli, specifically, low-loneliness participants showed greater activation in the right caudate and in the rIFG when viewing ‘unpleasant’ *s*ocial situations, but no difference in IFG activation was found for pleasant social situations (Cacioppo *et al.*, 2009). As the rIFG was found to be connected to both observed touch perception and to loneliness, we chose to examine its role and association with both.

In this study we focused on the way low- and high-loneliness individuals perceive social touch and on the way that activation of the rIFG may affect their reaction to observed positive affective touch between humans. First, we used a direct measure of attitudes toward touch by administering the social touch questionnaire (STQ) (Wilhelm *et al.*, 2001) and analyzed the three subscales proposed by Vieira *et al.* (2016): Dislike of physical touch, liking of familiar physical touch and liking of public physical touch. We hypothesized that lonely individuals would show less liking of social touch in the STQ, representing diminished attraction to social touch. To causally examine the role of the rIFG, we tested the impact of an anodal tDCS to the rIFG while participants observed pictures depicting humans touching or not touching, or inanimate objects touching or not touching. The participants were requested to identify whether the picture they were presented with displayed humans or inanimate objects. Half of the stimuli contained touch. The purpose of including inanimate objects in the study design was 2-fold. First, it was needed for the task itself so that touch could remain an implicit variable—the participants were asked to indicate if the image included objects or humans, and therefore touch was not mentioned explicitly. Second, this also allowed to test whether potential findings with regard to the impact of rIFG stimulation were specific to observing human interaction or to observing any physical contact.

Participants received either anodal stimulation or sham stimulation while performing the task. Based on the premise that positive valence stimuli serve as distractors and cause increased reaction times (Gupta *et al*., 2016). It was hypothesized that increased rIFG excitability would result, among the low-loneliness participants, in an increased emotional reaction toward social touch, which will lead to increased reaction time in the condition in which human touch was included as an implicit distractor. Furthermore, if lonely people have activated a self-preserving state, which keeps them focused on their selfish interests rather than on expecting positive social interaction (Cacioppo and Cacioppo, 2018), activating the rIFG should not impact their reaction time, as they would not be emotionally distracted by touch. We therefore hypothesized a difference in the emotional response following rIFG stimulation between lonely and non-lonely individuals, such that lonely individuals would be less emotionally distracted than non-lonely individuals.

## Methods

### Participants

Forty participants (18 males) participated in the study (ages 20–39, mean age = 25.16, s.d. = 3.72, median age = 25). The sample size was determined using an a priori sample size estimate for 0.8 power and 0.6 effect size, based on prior tDCS studies (Minarik *et al.*, 2016; Schroeder *et al.*, 2020). The analysis was a two-tailed *t*-test for differences between two dependent means with an alpha of 5%. It is noteworthy that while the calculation yielded a minimum sample size of 24, we decided to test our paradigm on a larger sample.

Participants received either course credit or payment for participating. All participants met the inclusion criteria according to brain stimulation protocols (Nitsche *et al.*, 2003; Bikson *et al.*, 2009). All participants had normal or corrected-to-normal vision and normal hearing and gave written informed consent prior to inclusion in the study. The study was approved by the University of Haifa Ethics Committee. A couple of weeks prior to the experiment, each participant completed the University of California Los Angeles (UCLA) loneliness scale III questionnaire (Russell, 1996) to assess level of loneliness as well as the STQ (Wilhelm *et al.*, 2001) to measure preferences concerning social touch. Three participants were excluded from the data analysis since they did not complete the UCLA loneliness questionnaire properly. Hence, the reported results are based on 37 participants (15 males).

### Stimuli, task and design

**Fig. 1.**
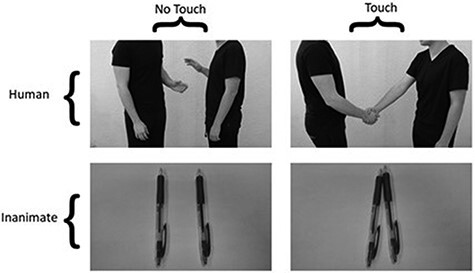
Photographs illustrating the four conditions.

The study used a variation of a task that was reported in a previous publication (Peled-Avron *et al.*, 2019). Participants completed a computerized task (using E-Prime 2.2 Psychological Software Tools for stimulus presentation and experimental control). The participants sat approximately 60 cm across from a 21″ flat screen monitor and were shown monochromatic images, all sized 6″ × 4″ (∼15 cm × 10 cm), landscape orientated with fixed luminance. The participants were presented with 80 images, 20 in each of the four conditions: human touch, human non-touch, inanimate touch and inanimate non-touch. The human touch condition contained photographs of various types of social touch, for example a hug or a handshake. The inanimate touch condition (control condition) included photographs of two everyday objects (without any commercial logo) touching each other and positioned in various ways. The other two conditions presented the same humans or objects in proximity but not touching. Inanimate objects and humans were photographed against a white background. All humans wore black clothing and were photographed from the shoulders down to avoid the confounding effects of facial expressions. Female subjects were presented with images of female humans, and male subjects were presented with images of male humans to avoid the confounding effects of romantic touch (See Figure 1).

### Procedure

A couple of weeks prior to completing the computerized task, participants completed a Hebrew version of the UCLA scale questionnaire (Russell, 1996). This version was translated into Hebrew using a cross-translation and validation process by two independent native speakers of both English and Hebrew.

The UCLA scale was initially developed in 1978 (Russell *et al.*, 1978) and has since been revised twice to improve its validity and reliability. In the current version the respondent is asked to rate the frequency of loneliness-related experiences. Each item is rated on a scale of 1 (never) to 4 (often), and after reversing the questions that relate to positive experiences a total loneliness score (20–80) is calculated. The median score in the UCLA scale in the study was 36 (s.d. = 10.95). Participants were classified into a high-loneliness group (UCLA > 36; *N* = 19 participants) or a low-loneliness group (UCLA ≤ 36; *N* = 18 participants) based on the median score, as was done in multiple past studies (Cacioppo *et al.*, 2015b, 2016). The mean loneliness scores in the high loneliness and low loneliness groups were 30.00 ± 3.99 and 47.47 ± 8.25, respectively.

The participants also completed a Hebrew version of the STQ (Wilhelm *et al.*, 2001). This version was translated into Hebrew using a cross-translation and validation process by two independent native speakers of both English and Hebrew. The STQ is a 20‐item self‐report measure of preferences regarding social touch. Items include 10 negative and 10 positive statements about social touch. The scores range from 0 to 80, with lower scores indicating a preference for social touch and higher scores indicating that social touch is perceived as unpleasant and is avoided in various contexts. For the purposes of this study, we also used the three subscales (Vieira *et al.*, 2016) and calculated them separately: (i) dislike of physical touch—a subscale that includes all 10 negative statements that reflect negative emotional reactions toward touch, including avoidance, feeling uncomfortable, stress, irritation or disgust when being touched in various situations; (ii) liking of familiar physical touch—a subscale that includes six statements that reflect positive emotions toward being touched by people I am familiar with and (iii) liking of public physical touch—a subscale that includes four statements that reflect positive emotions toward touch in general.

The study used a randomized, single-blind, sham-controlled, within-subject design. Each participant was invited to two sessions with a 1-week interval between them. Each session included either sham or anodal stimulation. The order of stimulations was counterbalanced across participants. The participants were not aware of the type of stimulation they received, while the experimenter was fully informed (see Cattaneo *et al.*, 2011; Peled-Avron *et al.*, 2019; Pisoni *et al.*, 2012 for similar procedures). Participants were debriefed after each session to confirm that they had not been able to distinguish between the sham and the stimulation conditions. After each session participants were asked the following question: ‘Do you think today you received the control or the test stimulation? Yes/No /I don’t know’. All the participants replied ‘I don’t know’ in both sessions. The task began 3 min after the onset of the stimulation. This period was selected since studies have shown that cortical excitability changes due to tDCS can be observed after 3 min of stimulation (Nitsche and Paulus, 2001; Nitsche and Bikson, 2017). The stimuli were presented in four blocks of 20 trials each, for a total of 80 trials. Blocks were randomized across stimulation conditions and participants. A block design was used to allow for short breaks, which were used to allow participants to rest and indicate if they were experiencing any discomfort. Three practice trials were included in the instructions phase of the experiment to ensure comprehension. Each block contained five trials from each condition (human no-touch, human touch, inanimate no-touch and inanimate touch). In two of the blocks the participants were instructed to press the space bar if they identified humans in the photo, and in the other two blocks they were instructed to press the space bar if they identified inanimate objects in the photograph. Each trial consisted of a fixation cross shown for 500 ms, followed by an image shown for up to 4000 ms. The image presentation concluded as soon as the participant responded. An inter-trial interval of a blank screen was presented for 400 ms. The reaction time was logged for each trial, as well as an indication of response accuracy (i.e. if the participant correctly identified humans or inanimate objects when presented).

### tDCS

tDCS was delivered using a battery-powered constant current stimulator (Magstim, Whitland, Wales, UK) via two saline-soaked sponge electrodes (experimental electrode: 25 cm^2^ 5 × 5, reference electrode: 35 cm^2^ 7 × 5). The electrodes were placed on the participant’s head and held using textile straps. A constant current of 1.5 mA was applied for 15 min. Participants performed the task during the stimulation or sham condition. The task duration was 10–12 min, including practice sessions and breaks. To ensure homogeneity of stimulation length, participants that concluded the task in less than 15 min were instructed to remain seated until the experimenter switched off the device at the end of the 15-min period.

Localization was established using the 10–20 electroencephalography system. During the stimulation condition, the anodal electrode served as the experimental electrode and was placed on the right IFG, which was determined to be the crossing point between T4-Fz and F8-Cz (Jacobson *et al.*, 2012).

The cathodal electrode served as the reference electrode and was placed above the left frontopolar cortex (Nitsche and Paulus, 2001). During the sham stimulation, the placement of electrodes was identical; however, the current was turned off 30 s after the beginning of the stimulation. In both conditions the current was turned on and off in a ramp-like fashion for a duration of 7 s (Nitsche *et al.*, 2003; Ambrus *et al.*, 2012). This produced a transient tingling sensation on the scalp that faded after a few seconds. This procedure ensures both anodal and sham conditions include the same sensation, which allows for successful blinding of participants to the stimulation condition (Gandiga *et al.*, 2006).

## Results

**Fig. 2.**
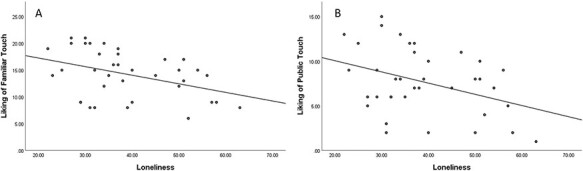
Lonelier individuals reported less liking of familiar physical touch (A) and less liking of public physical touch (B). Note that in the original questionnaire scoring, the items in the two subscales are reverse-scored. In our analysis the scores were not reversed, for the purpose of a more intuitive presentation.

Statistical analyses were performed using SPSS 25.0. We examined the correlations of the UCLA score with the three subscales of the STQ. A significant negative correlation was found with two subscales: liking of familiar physical touch [*r*(35) = −0.4, *P* = 0.01] and liking of public physical touch [*r*(35) = −0.38, *P* = 0.02], indicating that lonelier individuals reported less liking of familiar physical touch and public touch (see Figure 2). There was no significant correlation with the last subscale, dislike of physical touch [*r*_(35)_ = −0.12, *P* = 0.49] or with the total STQ score [*r*_(35)_ = −0.28, *P* = 0.09].

In order to examine baseline differences, we first calculated a mixed-design analysis of variance (ANOVA) for all the sham stimulation conditions, with the reaction time as the dependent variable. Touch condition (touch and no-touch) and image type (human and inanimate) served as the within-subject factors and loneliness group (high loneliness and low loneliness) as the between-subject factor. This analysis yielded a main effect for image type, with a faster reaction time to human images (*M* = 388.26, s.d. = 71.78) compared to inanimate images [*M* = 433.54, s.d. = 78.89; (*F*_(1,35)_ = 67.61, *P* < 0.001, η*_p_*^2^ = 0.66]. No other main effects or interactions were significant (*F* < 2.36, *P* > 0.13).

To test the influence of IFG stimulation, compared to sham, on the viewing of human images, a mixed-design ANOVA with the reaction time as the dependent variable was employed for the human images. Stimulation (sham and anodal) and touch condition (human touch and human no-touch) served as the within-subject factors and loneliness group (high loneliness and low loneliness) as the between-subject factor. This analysis yielded a significant interaction among stimulation, condition, and loneliness [*F*_(1,35)_ =  4.71, *P *= 0.04, η*_p_*^2^ = 0.12]. No other main effects or interactions were found [*F*_(1_,_35)_ < 3.66, *P* > 0.06].

**Fig. 3.**
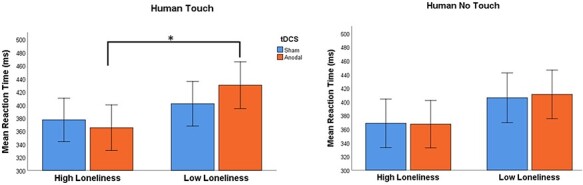
In the human images conditions, a significant interaction among stimulation, condition and loneliness scores was found; follow up t-test analysis showed that under anodal stimulation the reaction time of the low-loneliness group in the human touch condition was significantly higher compared to the high-loneliness group [Error bars: 95% confidence interval (CI)].

Follow-up *t*-tests showed a significant difference between the reaction times of the high-loneliness group and the low-loneliness group in the human touch condition under the anodal stimulation (See Figure 3). The reaction time of the low-loneliness group in the human touch condition (M0078 = 429.89, s.d. = 95.06) was significantly higher than that of the high-loneliness group in that condition [*M* = 365.13, s.d. = 48.13; *t*_(35)_ _=_ 2.636, *P*_corr_ = 0.048, *P* Bonferroni-corrected for multiple comparisons, Cohen’s *d* = 0.86]. No other *t*-tests were found to be significant (*t*_(35)_ < 1.74, *P* > 0.10); see Table 1 for details on the *t*-test analyses.

**Table 1.** Independent samples t-tests for the significant interaction among stimulation (sham and anodal), touch condition (human touch and human no-touch) and loneliness group (high loneliness and low loneliness)

	Loneliness	*N*	Mean	s.d.	*t*-test	*P*_corr_	Cohen’s *d*
RT human touch—sham	High	19	377.097	55.423	[*t*_(35)_ = 1.054, *P* = 0.299]	1	0.347
	Low	18	401.701	84.415			
RT human touch—anodal	High	19	365.127	48.135	[*t*_(35)_ = 2.636, *P* = 0.012]*	0.048*	0.867
	Low	18	429.889	95.061			
RT human no-touch—sham	High	19	368.439	51.463	[*t*_(35)_ = 1.495, *P* = 0.144]	0.576	0.492
	Low	18	405.797	95.321			
RT human no-touch—anodal	High	19	367.267	42.296	[*t*_(35)_ = 1.777, *P* = 0.095]	0.38	0.584
	Low	18	410.717	97.408			

*P*_corr_ = Bonferroni-corrected *P* value, *P < 0.05.

Paired *t*-tests of the difference in reaction times to human touch and to human no-touch images between sham *vs* anodal condition were not significant for the entire study population or when examining the two loneliness groups separately [*t*_(17)_ < 2.04, *P* > 0.05].

**Fig. 4.**
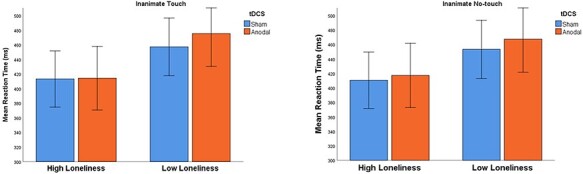
In the inanimate conditions, no significant main effects or interactions were found (Error bars: 95% CI).

To test if this finding was specific to human interaction, we repeated the analysis—this time with the inanimate objects’ images instead of the human images. A mixed-design ANOVA with the reaction time as dependent variable, with stimulation (sham and anodal) and touch condition (inanimate touch and inanimate no-touch) as the within-subject factors and loneliness group (high loneliness and low loneliness) as the between-subject factor, yielded no significant main effects or interactions [*F*_(1,35)_ < 3.36, *P* > 0.07]. Specifically, the interaction among stimulation, condition and loneliness was not significant [*F*_(1,35)_ = 0.23, *P *= 0.63, η*_p_*^2^ = 0.007]. The results of the analysis of the inanimate objects are presented in Figure 4.

As an additional control analysis, we probed the influence of gender. A mixed-design ANOVA with the reaction time as dependent variable was employed for the human images. Stimulation (sham and anodal) and touch condition (human touch and human no-touch) served as the within-subject factors and gender (male and female) served as the between-subject factor. This yielded no significant main effects or interactions [*F*_(1,35)_ < 1.96, *P* > 0.170]. In addition, we examined the influence of age. A mixed-design ANOVA with the reaction time as dependent variable was employed for the human images. Stimulation (sham and anodal) and touch condition (human touch and human no-touch) served as the within-subject factors and age (younger/older) served as the between-subject factor. This yielded no significant main effects or interactions [*F*_(1,35)_ < 1.44, *P* > 0.24]. Furthermore, we examined the influence of order of sham/anodal stimulation. A mixed-design ANOVA with the reaction time as dependent variable was employed for the human images. Stimulation (sham and anodal) and touch condition (human touch and human no-touch) served as the within-subject factors and order (sham first and anodal first) served as the between-subject factor. This yielded no significant main effects or interactions [*F*_(1,35)_ < 1.95, *P* > 0.17].

The average overall error rate (pressing human when an object was presented or pressing object when a human was presented) was 3.45% (s.d. = 4.47) and omission rate was 0%, translating to an accuracy rate of 96.55%. A mixed-design ANOVA with the error rate as dependent variable was employed. Stimulation (sham and anodal) and touch condition (human touch and human no-touch) served as the within-subject factors and loneliness group (high loneliness and low loneliness) as the between-subject factor. No significant main effects or interactions were found [*F*_(1,35)_ < 2.27, *P* > 0.14].

## Discussion

In this study, we set out to advance our knowledge about the behavioral and neural mechanisms that mediate responses to the observation of social touch among low- and high-loneliness individuals, examining the causal effect of rIFG anodal tDCS.

We initially examined specific subscales of the attitudes toward STQ (Vieira *et al.*, 2016) and we found that while loneliness was not correlated with dislike, or negative emotional response toward physical touch, it was negatively correlated with liking of familiar physical touch and liking of public physical touch. This signifies that lonely people do not necessarily avoid or dislike touch, however they express less liking of it. This finding sheds light on the way lonely people perceive positive social stimuli, and specifically social touch. It supports the hypothesis according to which lonely people are not drawn to social touch, as they may have entered a self-preserving state (Cacioppo and Cacioppo, 2018). According to this notion, loneliness is an indication of an environment that the individual cannot rely on for support. Therefore, the individual does not expect positive social interaction and will therefore not be emotionally drawn to positive social cues such as affective human touch. This might explain why the high-loneliness group reported less liking of social touch.

Our hypotheses with regard to the rIFG stimulation effect were partially confirmed. We demonstrated that an anodal tDCS stimulation of the rIFG resulted in a significant difference in reaction time between high- and low-loneliness individuals to human touch images, such that low-loneliness individuals showed a higher reaction time. This supports previous studies that showed the rIFG is an important part of an inferior frontoparietal OE network. The OE network was first established in the context of motor activity, as it has been repeatedly demonstrated that when one observes another performing a motor action it activates one’s own motor representations, which in turn results in an activation of motor areas of the brain where responses are prepared and executed (Thornton and Knoblich, 2006). In humans, the OE system has been identified in the IFG, the inferior parietal lobule (Iacoboni *et al.*, 2001; Lestou *et al.*, 2008), the superior temporal sulcus (Mainieri *et al.*, 2013) and the premotor cortex (Wade and Hammond, 2015). It is increasingly accepted that the OE system is involved not only in motor action observation but also in other social contexts such as the observation and execution of cognitions and emotions (Cacioppo and Cacioppo, 2012; Shamay-Tsoory *et al.*, 2019). It was further suggested that a simulation OE neural network for the perception of touch exists (Peled-Avron *et al.*, 2019; Schirmer and McGlone, 2019) and that this network involves an activation of past representations of social touch when observing someone else engaged in such positive affective touch. The results of this study support this suggestion, as a direct stimulation of the rIFG specifically modified reaction times to observed human touch. It is noteworthy that this response occurred solely when observing human social touch and was not found when viewing images of inanimate objects. Future studies should apply a combined approach of stimulation and imaging paradigms to further investigate the role of the rIFG in the perception and response to human touch, as well as investigate the connectivity of the rIFG with other simulation–observation network regions in the context of social touch observation.

The marked difference in reaction to human touch images between high- and low-loneliness individuals under anodal stimulation is in agreement with previous studies that showed the impact of IFG stimulation may be different for different populations. For example, anodal tDCS stimulation of the left IFG increased interpersonal motor resonance among individuals with low perspective taking ability (Fini *et al*., 2017) and anodal stimulation of the right IFG increased emotional response to observed social touch but only among individuals with low empathy (Peled-Avron *et al.*, 2019).

Our results advance the knowledge on possible neural correlates of loneliness. If a direct stimulation of the rIFG impacted low- and high-loneliness individuals differently in the response to the observation of human social touch, it could be suggested that the rIFG is related to the social impairments displayed by lonely individuals. Our results suggest that these impairments are not a product of hypoactivity of the rIFG, since if that were the case, excitation of the rIFG should have resulted in a normalized response. Our findings support previous studies that show impaired connectivity of the rIFG among lonely participants in the ventral attention network (Tian *et al.*, 2014), reduced feelings of loneliness among people who suffer from lesions to the rIFG (Cristofori *et al.*, 2019) and increased activity of the OE network among lonely people during social interaction (Saporta *et al.*, 2021a, preprint).

Our hypothesis with regard to the effect of rIFG anodal stimulation was only partially confirmed. While a large effect size (Cohen’s *d* = 0.86) was found in the comparison of the reaction time of high- and low-loneliness subjects to human touch images under anodal stimulation of the rIFG, which indicates that the difference may be substantial, anodal stimulation did not result in a main effect for touch condition (human touch–human no-touch), but only a significant interaction. It is therefore conceivable that the interaction that was found among loneliness, touch and tDCS stimulation is not attributable necessarily to increased emotional reaction as a result of the anodal stimulation. The IFG is also considered a primary part of neural pathways of inhibitory control and it was previously shown that anodal stimulation of the rIFG increased inhibitory control (Cai *et al.*, 2016; Zhang and Iwaki, 2019). Thus, anodal stimulation may have produced increased reaction time due to inhibitory processes. However, the difference between the high- and low-loneliness groups was uniquely found in the human touch condition and not in the human no-touch or the inanimate touch/no-touch conditions. This specific effect speaks against a general inhibitory process. It is also possible that the sample size was not large enough to detect a main effect, if it existed. Future studies should increase the sample size and include emotion-related questions and physiological measures in the design to correlate with the behavioral data in order to validate the task assumptions with regard to increased emotional reaction and to confirm the study findings.

It can be claimed that the finding that the overall reaction time to human images was faster when compared to inanimate images speaks against the assumption that human images create an emotional distraction. However, we propose that this finding is likely the result of the design of the stimuli. Specifically, the human figures were larger compared to the objects and had relatively smaller space around them compared to objects as can be seen in Figure 1. Prior research shows that reaction time is influenced by such factors so that reaction time is faster when the stimulus is larger (Osaka, 1976; Kosinski, 2008; Dureux *et al.*, 2021). It should also be noted that in the baseline analysis, under sham stimulation, we did not detect an interaction between loneliness and touch condition (human touch–human no-touch). It is possible that under sham stimulation the discrimination task was too simple, and therefore distraction by emotional stimuli was minimal. Instead, it could be that the salience of the human stimuli, which is more relevant from an evolutionary perspective captured the attention of the participants, resulting in faster reaction time (see for example, Yang *et al.*, 2012) and that only under anodal stimulation, which may have increased the emotional reaction to observed human touch among the low-loneliness group, the human touch images were distracting enough. Future studies could potentially increase the task difficulty, making it more likely to be impacted by emotional distraction.

The IFG is also involved in motor OE (Shamay-Tsoory *et al.*, 2019) and therefore its stimulation could result in an increase in attention to any perception of motion. When focusing on the IFG specifically, it appears that the left IFG is associated to a greater extent with motor OE (Iacoboni *et al.*, 1999; Gerardin *et al.*, 2000), whereas the rIFG is more associated with emotional observation execution and emotional empathy (Perry *et al.*, 2012; Wu *et al.*, 2018). The rIFG was also found to be related to tactile processing (Reed *et al.*, 2005; May *et al.*, 2014). As our research question revolved around emotional aspects of touch, we chose to investigate the role of the rIFG. Moreover, the specific effect we found demonstrates that it was the touch that elicited the differential response, as the human no-touch condition included pictures of humans in motion as well. This study used anodal tDCS stimulation compared to sham and did not use cathodal tDCS stimulation. We chose to do so based on prior research that demonstrated anodal stimulation consistently increased cortical excitability compared to sham, while conflicting findings exist with regard to the effect of cathodal stimulation (Dyke *et al.*, 2016; Thair *et al.*, 2017). Another limitation of this study is that we used a measurement of loneliness that was designed to measure chronic loneliness (Russell, 1996). Future studies may utilize additional measurements to assess the different impact of short-term *vs* more chronic loneliness.

In conclusion, in this study we showed that under anodal stimulation of the rIFG, there was a marked difference between high- and low-lonely individuals’ reaction to observed human touch. We suggest that anodal stimulation of the rIFG increased the emotional response toward observed positive social human touch, however impacting high- and low-loneliness individuals differently, with more impact on the low-loneliness group. Moreover, we show that the high-loneliness group reports less liking of positive social touch. This may help explain the perpetuation of loneliness, despite social opportunities that may be available to lonely people. From a clinical perspective, our study demonstrates that the lonely brain may respond differently even to direct neural manipulation. This study further advances our understanding of loneliness and may contribute to devising effective models of intervention that will break the perpetuation cycle and aid lonely individuals with their social function in society.
